# Cross-sectional analysis of adult fatty liver prevalence in Wenshan, China: an epidemiologic study using ultrasound and laboratory assessments

**DOI:** 10.3389/fcvm.2026.1571261

**Published:** 2026-01-30

**Authors:** Guangpin Zeng, Guanghong Li, Xinghong Lin, Shoude Yang, Linran Zeng

**Affiliations:** 1 Quality Control Department, The People's Hospital of Wenshan Prefecture, Wenshan, China; 2 General Practice, People's Hospital of Wenshan Prefecture, Wenshan, China; 3 Geriatrics and Gastroenterology Department, The First Affiliated Hospital of Kunming Medical University, Kunming, China

**Keywords:** epidemiology, hyperlipidemia, hyperlipidemia and liver injury, liver injury, non-alcoholic fatty liver disease (NAFLD), southwest China border, Wenshan region

## Abstract

**Background:**

The epidemiological profile of non-alcoholic fatty liver disease (NAFLD) in Wenshan City, a southwestern Chinese city with a population of 3.4 million, is not well-defined. This study aimed to examine the prevalence of NAFLD and its risk factors among healthy adults in Wenshan City.

**Methods:**

This study included 11,997 individuals who underwent health checkups at the People's Hospital of Wenshan Prefecture in 2022. Of these, 6,419 eligible adult subjects were analyzed. Each participant received abdominal ultrasonography and laboratory tests. Statistical analysis using R language was conducted to compare NAFLD prevalence, estimate odds ratios for NAFLD risk factors via logistic regression, and examine correlations between NAFLD and factors like age and gender. Additionally, the relationship between NAFLD, hyperlipidemia, liver injury, and the combination of hyperlipidemia and liver injury was examined.

**Results:**

Among the 6,419 patients studied, the average age was 44.8 years (SD = 14.1), with a gender distribution of 59.4% males (3,815) and 40.6% females (2,604). The overall prevalence of NAFLD was 33.3% (2,138/6,419), with 39.6% (1,511/3,815) in males and 24.1% (627/2,604) in females. The average age of NAFLD patients was 47.9 ± 13.3 years, with a tendency to rise as age increased. The study found a high prevalence of hyperlipidemia (40.4%), liver injury (32.3%), and their combination (17.3%) in Wenshan adults, with higher rates in males than females. Prevalence increased with age until 60, after which it declined in males but rose in females for NAFLD. In the NAFLD population, hyperlipidemia was prevalent in 63.9% (69.1% males, 51.5% females), liver injury in 43.4% (51.0% males, 25.2% females), and both conditions in 31.3% (38.3% males, 14.4% females).

**Conclusion:**

The findings highlight significant associations between fatty liver, gender, age, and related health conditions. This finding suggests that special attention should be given to the prevention and management of NAFLD in border regions and integrated management.

## 1 Introduction

Wenshan City, located in the Wenshan Zhuang and Miao Autonomous Prefecture, is known for its diverse ethnic composition and varying lifestyle factors. Since the restoration of construction in the war zone, the area has experienced local economic and social development. Traditional foods have gradually been replaced by more convenient modern options, including the rising popularity of instant foods (such as fast food and baked goods) and the addition of sugary preparations to many instant products. Diets have increasingly become rich in energy, proteins, fats, saturated, and polyunsaturated fatty acids. This dietary shift may correlate with a rise in the incidence and severity of chronic diseases, potentially contributing to the prevalence of non-alcoholic fatty liver disease (NAFLD) (1–4). Non-alcoholic fatty liver disease (NAFLD), a significant global health issue, is increasingly prevalent and linked to metabolic disorders like obesity, cardiovascular disease, and liver cancer (5–7). NAFLD is marked by fat accumulation in liver cells without significant alcohol intake or infectious liver disease and is associated with metabolic syndromes such as hyperlipidemia and liver function abnormalities (8). It encompasses a spectrum of conditions characterized by chronic hepatic fat accumulation and non-bacterial inflammation, ranging from simple steatosis to cirrhosis due to ongoing liver damage and repair cycles. Hepatocellular carcinoma can develop in certain individuals with cirrhosis, adding societal and familial burdens (9–11). Ultrasonography is frequently employed to diagnose and assess fatty liver disease, while laboratory tests evaluate the extent of liver damage. Diagnostic methods offer crucial insights into the prevalence of fatty liver disease and its metabolic correlates (12–14). Prior research indicates a significant link between hyperlipidemia and the progression of fatty liver disease (15, 16). Moreover, liver function tests are essential for evaluating liver injury and functional abnormalities related to fatty liver disease. Abnormal liver function often signals liver damage and aids in assessing the severity of the condition (17, 18). The interplay between fatty liver disease and liver injury can escalate to more severe conditions like non-alcoholic steatohepatitis (NASH) and cirrhosis, significantly impacting patient health and the healthcare system (19). The prevalence of fatty liver disease has increased across various populations due to enhanced economic conditions and dietary changes, with NAFLD affecting 29.6% of individuals in Asia, potentially exceeding rates in Western populations (20, 21). Non-alcoholic fatty liver disease (NAFLD) is a significant public health issue in Asia, affecting 34% of the population (13, 22). The prevalence of NAFLD is rising in China, particularly in regions with varying socio-economic and lifestyle traits. In southwest China, Chengdu reports a prevalence of 12.5%, while Chongqing has a higher rate of 28.5% (6, 23). In other regions, the prevalence is 15.0% in Shanghai (east China), 17.0% in Guangdong (south China), and 24.5% in central China. Approximately 20%–30% of normal livers develop NAFLD, with 7%–30% advancing to NASH, 6%–7% directly to NAFLD-HCC, and 35%–47% to NASH with fibrosis or cirrhosis, of which 7%–13% progress to NAFLD-HCC (24). NAFLD is a leading cause of HCC, showing an 11.5-fold increase in HCC prevalence among NASH patients in the United States. Globally, 25%–40% of patients with NAFLD have cardiovascular disease, with China at a much higher level. NAFLD is linked to a higher long-term risk of both fatal and nonfatal cardiovascular events (20, 25). Due to its often-undiagnosed nature and limited monitoring for hepatocellular carcinoma (HCC), NAFLD-related HCC is typically detected at an advanced stage, making curative treatment difficult (26). Worldwide, the age-standardized prevalence of NAFLD has risen, and the prevalence of NASH-induced HCC increases with age. NAFLD significantly contributes to the rising incidence of hepatocellular carcinoma (HCC), imposing substantial societal and economic burdens (27, 28). Effective screening and management strategies are crucial to mitigate HCC risk (29). In the United States, NAFLD prevalence is expected to rise by 63% by 2030, with the median age increasing from 50 to 55 years (30). The annual direct healthcare costs for NAFLD are approximately $103 billion, equating to $1,613 per patient. Projections based on obesity suggest that NASH-related healthcare costs per patient could reach $6,968 by 2039. In Germany, France, Italy, and the United Kingdom, the annual cost for NAFLD patients is about €35 billion, ranging from €354 to €1,163 per patient (31, 32)^.^ In the United States, liver cancer significantly increases healthcare costs, with an annual expenditure of $17,278 ± $5,726 (33). In developing regions like Asia, research from Hong Kong indicates that NAFLD's progression to NASH will become a major clinical and economic challenge over the next two decades, particularly affecting the elderly and those with advanced disease stages (34, 35). In Wenshan, the prevalence of fatty liver remains unreported, and its risk factors, including associations with hyperlipidemia and liver injury, are not well understood. This study aimed to examine the epidemiological traits of fatty liver and its association with hyperlipidemia and liver injury among adults in Wenshan, a frontier region in China. Utilizing ultrasound and laboratory data from healthy adults, the research seeks to offer a scientific foundation for preventing and treating NAFLD in the area.

## 2 Methods

### 2.1 Study population

**Figure 1:**
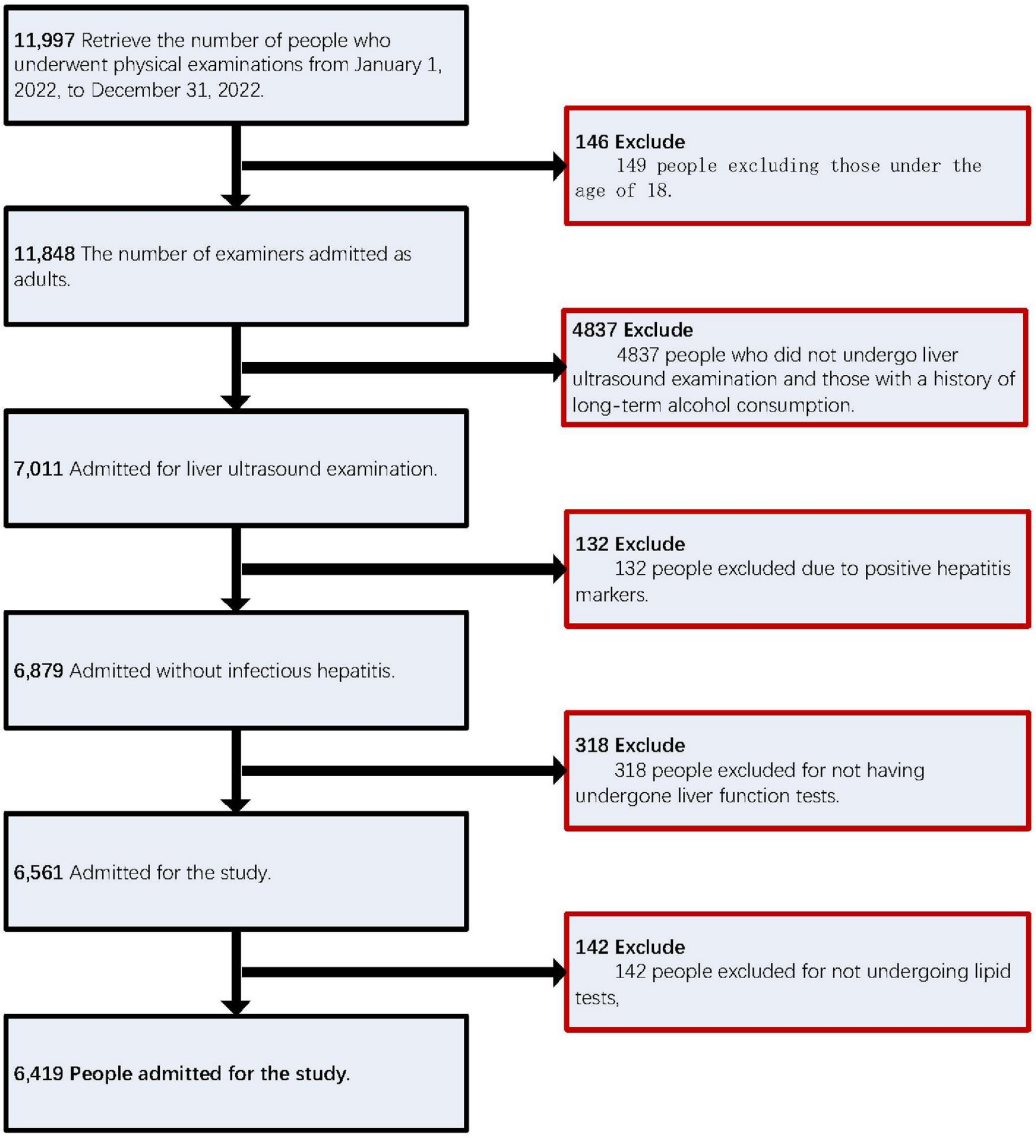
Shows the numerical ranking of the study population.

This study utilized data from adults who underwent routine physical examinations at the Physical Examination Center of the People's Hospital of Wenshan Prefecture between January 1 and December 31, 2022, either voluntarily or through employer-organized programs. Initially, 11,997 cases were included. Participants were excluded based on the following criteria: (1) age under 18 years; (2) incomplete abdominal ultrasound examination; (3) long-term excessive alcohol consumption (defined as >140 g/week for men and >70 g/week for women); (4) history of viral hepatitis or malignant liver cancer; or (5) missing key laboratory data. Following data cleaning, 6,419 eligible participants were included in the final analysis (Figure 1).

The study protocol was approved by the Ethics Committee of the People's Hospital of Wenshan Prefecture and authorized by the hospital's medical administration department. The Information Department assisted in the extraction and organization of the physical examination data. All researchers signed a confidentiality agreement to protect participant privacy and rights. The study received relevant guidance from experts at the Yang Hongju Expert Workstation in Wenshan Prefecture. All participants provided written informed consent in accordance with national regulations and institutional requirements.

It should be noted that this study relied on a physical examination database from the southwestern border region of China, which routinely collects only clinical and laboratory parameters. Therefore, several important lifestyle and anthropometric variables -such as body mass index (BMI), waist circumference, dietary habits, physical activity, smoking status, and socioeconomic factors -were not systematically available for this cohort. We acknowledge that this limitation may affect the interpretation of risk associations.

### 2.2 Ultrasound examination

Ultrasonography was used to determine the fatty liver condition. The operating physician, qualified in diagnostic ultrasound, was unaware of the study design. A high-definition diagnostic color ultrasound system and probe (Myers Mindray Colour Doppler Ultrasound System Resona I9T) was used for the examination. The criteria encompassed ultrasound features such as enhanced near-field hepatic echoes with gradual far-field attenuation, poor visualization of intrahepatic luminal structures, mild to moderate hepatomegaly with rounded edges, and color Doppler ultrasound indicating reduced or poorly visualized hepatic blood flow signals without intrahepatic vascular abnormalities (18, 36).

### 2.3 Biochemical measurements

Blood samples were collected in the morning following a minimum 8-hour fasting period. Variables collected included age, sex, lipid levels (TC, TG, LDL-C, HDL-C), liver function parameters (ALT, AST, GGT, total protein, globulin, albumin, albumin-globulin ratio, alkaline phosphatase, total bilirubin, direct bilirubin, indirect bilirubin, serum total bile acids), blood glucose (FBG), and uric acid (UA), bilirubin (total, direct, indirect), serum total bile acids, blood glucose (FBG), uric acid (UA), and viral hepatitis markers including Hepatitis B Virus Surface Antigen (HBsAg), Surface Antibody (HSAg), Core Antibody (HBsCoA), and e Antibody (HBsE Antibody). Hepatitis B Virus Surface Antigen (HBsAg), Surface Antibody (anti-HBs), Core Antibody (anti-HBc), e Antibody (anti-HBe), and e Antigen (anti-HBe). Hepatitis B Virus e Antigen (HBeAg). The tests were conducted using a Siemens ADVIA 2400 Automated Biochemical Analyzer. Infectious diseases were detected using an AI KANG DR6660-4 automated time-resolved immunoassay analyzer.

### 2.4 Definition of variables

#### 2.4.1 Hyperlipidaemic state

Hyperlipidemia is defined according to the NCEP-ATP III guidelines and the 2016 Chinese Guidelines for Adult Hyperlipidemia Prevention and Treatment. Hyperlipidemia was characterized by triglycerides (TG) ≥ 2.26 mmol/L, low-density lipoprotein cholesterol (LDL-C) ≥ 4.14 mmol/L, high-density lipoprotein cholesterol (HDL-C) < 1.04 mmol/L, or total cholesterol (TC) ≥ 6.22 mmol/L (37, 38).

#### 2.4.2 Liver function status

Abnormal liver function, indicative of liver injury, is diagnosed by elevated enzyme levels: alanine aminotransferase (ALT) ≥ 50 IU/L, aspartate aminotransferase (AST) ≥ 40 IU/L, and/or gamma-glutamyltransferase (GGT) elevation. Gamma-glutamyl transferase (GGT) is considered elevated at levels of 60 IU/L or higher, total bilirubin (TBIL) is elevated above 17.1 μmol/L, and direct bilirubin (DBIL) is elevated above 6.8 μmol/L. Criteria for determining liver injury: liver injury is defined as any one of the liver enzymes exceeding the upper limit of the lowest value (39).

#### 2.4.3 Glycemic status

In this study, glycemic status was categorized as normoglycemia for fasting blood glucose (FBG) levels below 6.1 mmol/L, and impaired fasting glucose (IFG) for FBG levels between 6.1 mmol/L and 7.0 mmol/L. Diabetes mellitus (DM) was characterized by a fasting blood glucose (FBG) level of 7.0 mmol/L or higher (40, 41).

#### 2.4.4 Hyperuricemia (Hua)

According to the “China Multidisciplinary Expert Consensus on the Diagnosis and Treatment of Diseases Associated with Hyperuricemia (2023 Edition)”, in adults with a normal purine diet, irrespective of sex, fasting blood uric acid level exceeds 420 *μ*mol/L (42).

#### 2.4.5 Dyslipidemia status & liver injury status

The presence of the above hyperlipidemia characteristics and the simultaneous presence of the above liver injury characteristics.

### 2.5 Statistical analysis

Statistical analyses were performed using R and Wind Rose software. A multivariable logistic regression model was used to assess independent risk factors for NAFLD. A p-value below 0.05 was deemed statistically significant.

## 3 Results

### 3.1 Baseline features of general population

The profile of the 6,419 eligible medical examiners was 59.4% male (3815/6419) and 40.6% female (2604/6419). The mean age of individuals with NAFLD (47.9 ± 13.3 years) was significantly higher than that of the non-NAFLD group (43.2 ± 14.2 years), with a statistically significant difference (*P* < 0.001). The prevalent age of NAFLD was 45.6 ± 13.0 years in men and 53.3 ± 12.2 years in women, which was significantly different compared to the non-NAFLD population (*P* < 0.001). The prevalence of NAFLD in the general population undergoing physical examinations was 9.6%, 31.2%, 41.7%, 17.5%, and 17.5% for individuals aged 60 years. In males, the prevalence was 12%, 36.8%, 37.8%, and 13.4%, while in females, it was 3.7%, 17.7%, 51.2%, and 27.4% for the same age group. In the general population, NAFLD prevalence was 63.9% among those with hyperlipidemia (69.1% in males, 51.5% in females), 43.4% among those with liver injury (51% in males, 25.2% in females), and 31.3% among those with both hyperlipidemia and liver injury (38.3% in males, 14.4% in females). The NAFLD population exhibited significantly higher levels of total bilirubin (TBil), direct bilirubin (DBil), indirect bilirubin (IBil), alkaline phosphatase (AKP), bile acids (TBA), urea nitrogen (BUN), and blood creatinine (CREA). Additionally, there was a higher prevalence of altered gender distribution, increased age, elevated white globe ratio (A/G), alanine aminotransferase (ALT), aspartate aminotransferase (AST), gamma globulin (GGT), total cholesterol (TC), triglycerides (TG), low-density lipoprotein cholesterol (LDL), fasting blood glucose (FBG), and uric acid (UA) levels, while high-density lipoprotein cholesterol (HDL) levels were significantly lower (*P* < 0.001) (Table 1).

**Table 1:** Comparison of baseline characteristics between participants with and without NAFLD, overall and by sex.

Characteristics	Total			Male			Female		
	NAFLD	Non-NAFLD	*p*	NAFLD	Non-NAFLD	*p*	NAFLD	Non-NAFLD	*p*
Age (years)	47.9 ± 13.3	43.2 ± 14.2	<0.001	45.6 ± 13.0	42.2 ± 14.7	<0.001	53.3 ± 12.2	44.4 ± 13.6	<0.001
Age (years)			<0.001			<0.001			<0.001
<30	205 (9.6)	913 (21.3)		182 (12)	561 (24.3)		23 (3.7)	352 (17.8)	
30–45	667 (31.2)	1482 (34.6)		556 (36.8)	835 (36.2)		111 (17.7)	647 (32.7)	
45–60	892 (41.7)	1341 (31.3)		571 (37.8)	607 (26.3)		321 (51.2)	734 (37.1)	
>60	374 (17.5)	545 (12.7)		202 (13.4)	301 (13.1)		172 (27.4)	244 (12.3)	
Lipid status			<0.001			<0.001			<0.001
Dyslipidemia status	1367 (63.9)	1226 (28.6)		1044 (69.1)	854 (37.1)		323 (51.5)	372 (18.8)	
Normal lipid status	771 (36.1)	3055 (71.4)		467 (30.9)	1450 (62.9)		304 (48.5)	1605 (81.2)	
Liver function status			<0.001			<0.001			<0.001
Liver injury	928 (43.4)	1147 (26.8)		770 (51)	876 (38)		158 (25.2)	271 (13.7)	
Normal liver function	1210 (56.6)	3134 (73.2)		741 (49)	1428 (62)		469 (74.8)	1706 (86.3)	
Dyslipidemia status and Liver injury status		<0.001			<0.001			<0.001	
Dyslipidemia &Liver injury status	669 (31.3)	441 (10.3)		579 (38.3)	377 (16.4)		90 (14.4)	64 (3.2)	
No simultaneous dyslipidemia and liver injury	1469 (68.7)	3840 (89.7)		932 (61.7)	1927 (83.6)		537 (85.6)	1913 (96.8)	
TP (g/L)	76.9 ± 4.1	76.3 ± 4.2	<0.001	76.7 ± 4.0	75.9 ± 4.2	<0.001	77.5 ± 4.2	76.8 ± 4.2	<0.001
GLB (g/L)	29.1 ± 3.7	28.7 ± 3.6	<0.001	28.5 ± 3.5	27.8 ± 3.6	<0.001	30.7 ± 3.7	29.7 ± 3.4	<0.001
ALB (g/L)	47.8 ± 2.6	47.6 ± 2.7	0.025	48.2 ± 2.5	48.1 ± 2.8	0.32	46.8 ± 2.6	47.1 ± 2.6	0.025
A/G Ratio	1.7 ± 0.2	1.7 ± 0.3	0.001	1.7 ± 0.2	1.8 ± 0.3	<0.001	1.5 ± 0.2	1.6 ± 0.2	<0.001
ALT (U/L)	33.8 ± 25.8	21.9 ± 22.4	<0.001	37.0 ± 28.3	26.0 ± 27.2	<0.001	26.0 ± 15.9	17.2 ± 13.6	<0.001
AST (U/L)	26.1 ± 16.3	21.8 ± 11.7	<0.001	27.0 ± 17.8	23.4 ± 13.4	<0.001	23.8 ± 11.8	19.9 ± 9.0	<0.001
GGT (U/L)	59.0 ± 64.5	33.2 ± 36.8	<0.001	67.7 ± 70.5	42.8 ± 43.3	<0.001	38.3 ± 39.8	22.1 ± 22.8	<0.001
TBil (μmol/L)	11.6 ± 5.5	11.7 ± 6.0	0.394	12.2 ± 5.6	13.0 ± 6.5	<0.001	10.0 ± 4.7	10.2 ± 5.0	0.645
DBil (μmol/L)	4.4 ± 1.7	4.5 ± 1.9	0.03	4.6 ± 1.6	4.9 ± 2.0	<0.001	4.0 ± 1.7	4.1 ± 1.6	0.223
IBil (μmol/L)	7.1 ± 4.0	7.2 ± 4.4	0.804	7.6 ± 4.2	8.1 ± 4.8	<0.001	6.1 ± 3.4	6.1 ± 3.6	0.93
ALP (U/L)	83.8 ± 22.9	76.0 ± 22.8	<0.001	82.8 ± 21.5	78.8 ± 20.9	<0.001	86.1 ± 25.8	72.7 ± 24.5	<0.001
TBA (μmol/L)	3.5 ± 5.6	3.2 ± 6.0	0.087	3.5 ± 4.5	3.8 ± 7.8	0.179	3.4 ± 7.6	2.5 ± 2.4	<0.001
BUN (mmol/L)	5.0 ± 1.3	4.9 ± 1.4	0.011	5.1 ± 1.3	5.1 ± 1.5	0.141	4.8 ± 1.3	4.6 ± 1.3	0.001
CREA (μmol/L)	79.2 ± 17.1	76.8 ± 19.3	<0.001	85.6 ± 14.7	87.9 ± 18.6	<0.001	63.7 ± 11.8	63.8 ± 9.5	0.797
TC (mmol/L)	5.3 ± 1.0	4.9 ± 1.0	<0.001	5.3 ± 1.0	4.9 ± 0.9	<0.001	5.3 ± 1.1	4.9 ± 1.0	<0.001
TG (mmol/L)	3.0 ± 2.9	1.6 ± 1.7	<0.001	3.2 ± 3.1	1.9 ± 1.9	<0.001	2.4 ± 2.3	1.3 ± 1.3	<0.001
HDL-C (mmol/L)	1.2 ± 0.3	1.4 ± 0.4	<0.001	1.1 ± 0.3	1.3 ± 0.3	<0.001	1.3 ± 0.3	1.6 ± 0.3	<0.001
LDL-C (mmol/L)	3.1 ± 0.9	3.0 ± 0.8	<0.001	3.1 ± 0.9	3.0 ± 0.8	<0.001	3.2 ± 0.9	2.9 ± 0.8	<0.001
FBG (mmol/L)	5.7 ± 1.6	5.1 ± 1.1	<0.001	5.7 ± 1.7	5.2 ± 1.3	<0.001	5.7 ± 1.3	5.0 ± 0.8	<0.001
UA (μmol/L)	403.2 ± 93.9	346.6 ± 89.9	<0.001	426.4 ± 89.3	395.4 ± 81.5	<0.001	347.3 ± 80.2	289.7 ± 61.3	<0.001

TP, Total Protein; ALB, Albumin; GLB, Globulin; A/G Ratio, Albumin/Globulin Ratio; ALT, Alanine Transaminase; AST, Aspartate Aminotransferase; GGT, Gamma-Glutamyl Transferase; TBIL, Total Bilirubin; DBIL, Direct Bilirubin; IBil, Indirect Bilirubin; ALP, Alkaline Phosphatase; TBA, Total Bile Acid; BUN, Blood Urea Nitrogen; CREA, Creatinine; TC, Total Cholesterol; TG, Triglyceride; HDL-C, High-Density Lipoprotein Cholesterol; LDL-C, Low-Density Lipoprotein Cholesterol; FBG, Fasting Blood Glucose; UA, Uric Acid.

### 3.2 Nafld prevalence in individuals undergoing physical examinations

**Figure 2:**
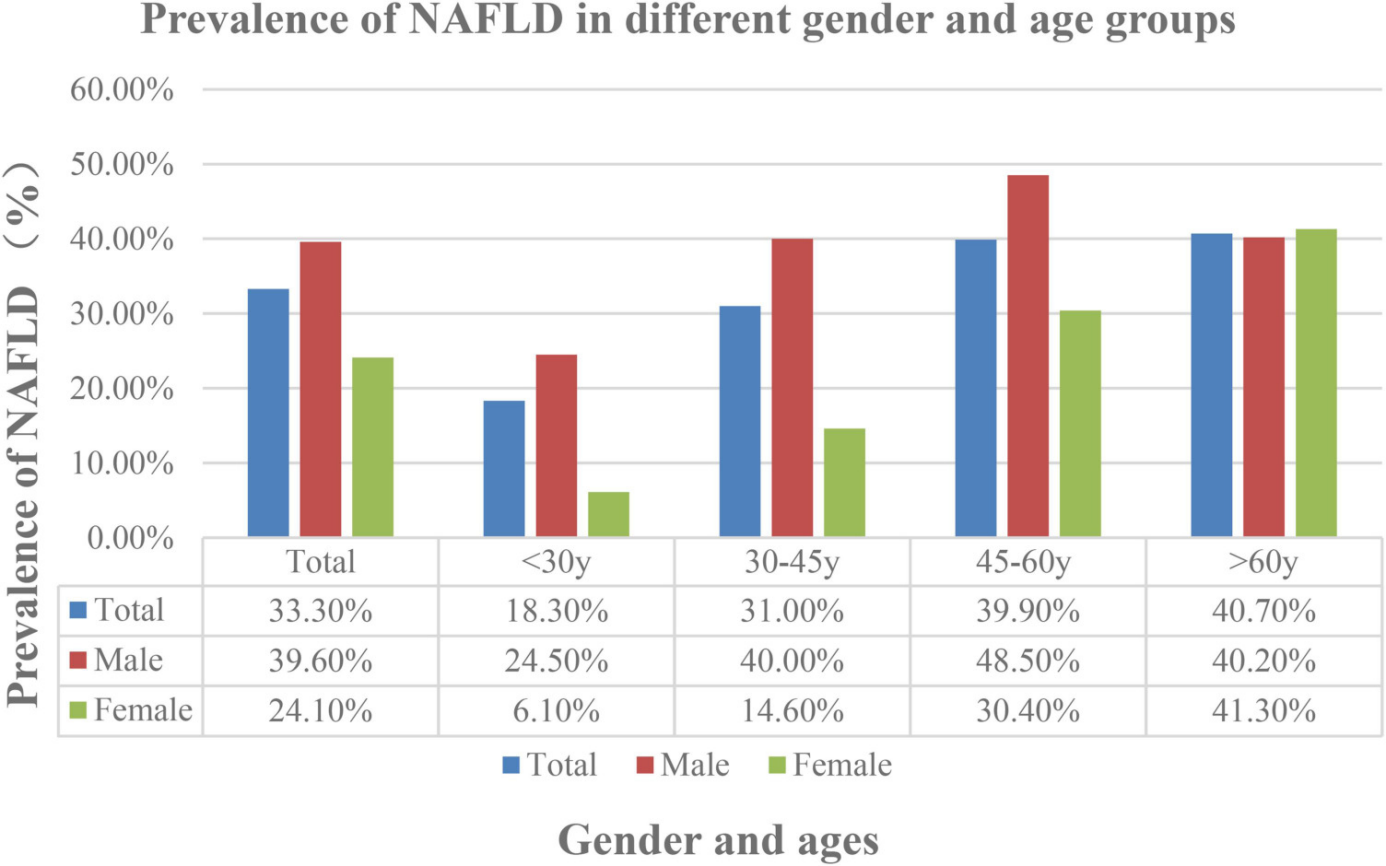
Prevalence of Non-alcoholic fatty liver disease (NAFLD) in different gender and ages.

Among 6,419 individuals, 2,138 were diagnosed with NAFLD, resulting in an overall prevalence of 33.3%, with 39.6% in men and 24.1% in women. The prevalence of NAFLD in the physical examination population rose from 18.3% in individuals under 30%–40.7% in those over 60. NAFLD prevalence in males rises from 24.5% at age 30 to a peak of 48.5% between ages 45–60, then slightly declines to 40.2% after age 60. For women, the prevalence rate starts at 6.10% for those under 30 and gradually increases with age, peaking at 41.3% for those over 60 (Figure 2).

### 3.3 NAFLD-related risk factors and their correlation with hyperlipidaemia, liver injury, hyperlipidaemia and liver injury

#### 3.3.1 Univariate logistic regression analysis

In individuals with NAFLD, variables including Non-NAFLD, Female, Age, TP, GLB, ALB, A/G ratio, ALT, AST, GGT, ALP, TBA, TBil, DBil, IBil, BUN, Cr, TC, TG, HDL-C, LDL-C, FBG, and UA showed significant associations with lipid status, with *P*-values < 0.05, and many being <0.001. This indicates a significant relationship between each variable and hyperlipidemia status. The OR values indicating a positive correlation were 4.42 for Non-NAFLD, 2.72 for Female, 1.31 for A/G Ratio, 1.17 for DBil, and 67.43 for HDL-C. Conversely, TC, TG, LDL-C, and FBG had OR values <1, indicating a negative correlation. Other laboratory indicators had OR values close to 1, suggesting weak correlations.

In NAFLD individuals, excluding BUN and LDL-C, variables including Non-NAFLD, Female, Age, TP, GLB, ALB, A/G ratio, ALT, AST, GGT, ALP, TBA, TBil, DBil, IBil, Cr, TC, TG, HDL-C, FBG, and UA were significantly associated with liver injury status, with many *P*-values < 0.001 and all <0.05. he OR values for Non-NAFLD, Female, and HDL-C were 2.1, 3.85, and 2.34, respectively, demonstrating a positive correlation. However, OR values for ALB, A/G Ratio, AST, TBil, DBil, IBil, TC, TG, and other laboratory indicators were <1, indicating a negative correlation. Other OR values were close to 1, suggesting weak correlations.

In NAFLD individuals, variables including Non-NAFLD, Female, TP, ALB, ALT, AST, GGT, ALP, TBA, TBil, DBil, IBil, Cr, TC, TG, HDL-C, LDL-C, FBG, and UA were significantly associated with the combined status of hyperlipidemia and liver injury, with *P*-values < 0.05, many being <0.001, excluding Age, GLB, A/G ratio, and BUN. R values for Non-NAFLD, Female, and HDL-C were 3.97, 5.32, and 22.76, respectively, indicating a positive correlation. Conversely, OR values for DBil, TC, TG, LDL-C, and FBG were <1, indicating a negative correlation. Other OR values were close to 1, suggesting weak correlations.

Non-NAFLD, Female, and HDL-C exhibited wide confidence intervals across all three statuses, with upper limits far greater than 1. This suggests a significant positive correlation between Non-NAFLD, Female, HDL-C, and the presence of hyperlipidemia, liver injury, and their combination in individuals with NAFLD.

In summary, from a statistical perspective, the results in the table indicate that most variables have significant impacts under different variable conditions. Notably, gender, NAFLD, and various lipid and liver function-related indicators are closely associated with hyperlipidemia, liver injury, and their combined complications (Table 2).

**Table 2:** Univariate logistic regression analyses of factors associated with dyslipidemia, liver injury, and their Co-occurrence.

Factor	Dyslipidemia Status	Liver Function Status	Dyslipidemia & Liver Injury
	OR (95% CI)	OR (95% CI)	OR (95% CI)
NAFLD (Yes vs. No)	4.42 (3.96–4.93)*	2.10 (1.88–2.34)*	3.97 (3.47–4.54)*
Female (vs. Male)	2.72 (2.44–3.03)*	3.85 (3.41–4.35)*	5.32 (4.45–6.36)*
Age (years)	0.98 (0.98–0.99)*	1.01 (1.01–1.01)*	1.00 (1.00–1.01)
ALT (U/L)	0.97 (0.97–0.97)*	0.92 (0.92–0.93)*	0.95 (0.94–0.95)*
AST (U/L)	0.97 (0.97–0.98)*	0.87 (0.87–0.88)*	0.94 (0.93–0.94)*
GGT (U/L)	0.98 (0.98–0.98)*	0.94 (0.94–0.94)*	0.96 (0.96–0.96)*
TC (mmol/L)	0.40 (0.38–0.43)*	0.83 (0.79–0.88)*	0.53 (0.50–0.57)*
TG (mmol/L)	0.06 (0.05–0.07)*	0.82 (0.79–0.84)*	0.60 (0.57–0.63)*
HDL-C (mmol/L)	67.43 (53.01–85.76)*	2.34 (2.00–2.75)*	22.76 (17.38–29.80)*
LDL-C (mmol/L)	0.56 (0.52–0.59)*	0.96 (0.91–1.02)	0.71 (0.66–0.77)*
FBG (mmol/L)	0.65 (0.62–0.69)*	0.90 (0.87–0.94)*	0.81 (0.78–0.84)*
UA (μmol/L)	0.99 (0.99–0.99)*	0.99 (0.99–0.99)*	0.99 (0.99–0.99)*

Results from three separate univariate logistic regression models are shown. OR, Odds Ratio; CI, Confidence Interval.

* *P* < 0.05.

#### 3.3.2 Multivariate logistic regression analysis of fatty liver risk factors based on gender

A multivariate logistic regression analysis was performed to determine key factors affecting non-alcoholic fatty liver disease (NAFLD) across various gender groups, including the overall population, males, and females. The analysis included variables such as lipid status, liver function status, the combined status of hyperlipidemia and liver injury, age, ALT, AST, GGT, and biochemical indicators such as hyperuricemia. Significance was assessed using OR values, 95% CI, and *P*-values. The results are as follows (Table 3).

**Table 3:** Multivariable logistic regression analysis of risk factors for nAFLD, stratified by gender.

Factor	Category	Total (*n* = 6,419)	Male (*n* = 3,815)	Female (*n* = 2,604)
		OR (95% CI)	OR (95% CI)	OR (95% CI)
**Dyslipidemia**	Normal (Ref)	1	1	1
	Present	**4.42 (3.96–4.93)**	**3.80 (3.31–4.36)**	**4.58 (3.78–5.56)**
**Liver Injury**	No (Ref)	1	1	1
	Yes	**2.10 (1.88–2.34)**	**1.69 (1.49–1.93)**	**2.12 (1.70–2.65)**
**Age Group**	≤30 years (Ref)	1	1	1
	30–45 years	**0.50 (0.42–0.60)**	**0.49 (0.40–0.59)**	**0.38 (0.24–0.61)**
	45–60 years	**0.34 (0.28–0.40)**	**0.34 (0.28–0.42)**	**0.15 (0.10–0.23)**
	>60 years	**0.33 (0.27–0.40)**	**0.48 (0.38–0.62)**	**0.09 (0.06–0.15)**
**ALT (U/L)**	≤50 (Ref)	1	1	1
	>50	**0.22 (0.18–0.26)**	**0.26 (0.21–0.32)**	**0.21 (0.13–0.32)**
**GGT (U/L)**	≤60 (Ref)	1	1	1
	>60	**0.33 (0.29–0.38)**	**0.40 (0.34–0.46)**	**0.32 (0.23–0.44)**
**TG (mmol/L)**	≤2.26 (Ref)	1	1	1
	>2.26	**0.21 (0.19–0.24)**	**0.26 (0.23–0.30)**	**0.18 (0.14–0.22)**
**HDL-C (mmol/L)**	≤1.04 (Ref)	1	1	1
	>1.04	**3.66 (3.22–4.15)**	**2.80 (2.42–3.23)**	**5.42 (4.05–7.26)**
**FBG (mmol/L)**	<6.1 (Ref)	1	1	1
	6.1–7.0	**0.32 (0.25–0.40)**	**0.47 (0.35–0.64)**	**0.16 (0.11–0.24)**
	>7.0	**0.22 (0.18–0.28)**	**0.33 (0.25–0.43)**	**0.11 (0.07–0.17)**
**UA (μmol/L)**	<420 (Ref)	1	1	1
	≥420	**0.36 (0.32–0.41)**	**0.53 (0.47–0.61)**	**0.13 (0.09–0.18)**

All models are adjusted for other variables listed in the table. OR, Odds Ratio; CI, Confidence Interval. Statistically significant results (*P* < 0.05) are presented in bold.

##### 3.3.2.1 Lipid status (dyslipidemia status)

Individuals with abnormal lipid profiles exhibited a significantly higher risk of NAFLD compared to those with normal lipid levels, with odds ratios of 4.42 for the overall population, 3.8 for males, and 4.58 for females (all *P* < 0.001). The increased risk in females indicates that dyslipidemia more significantly affects NAFLD risk in women.

##### 3.3.2.2 Liver function status

Individuals with liver damage exhibited a significantly higher risk of NAFLD compared to those with normal liver function (overall OR = 2.1; males OR = 1.69; females OR = 2.12; all *P* < 0.001). Females showed a stronger correlation between liver damage and NAFLD risk.

##### 3.3.2.3 Age

As age increases, the risk of NAFLD decreases significantly (overall population *P* < 0.001). The decline was more pronounced in males, while changes in females were relatively gradual.

##### 3.3.2.4 Liver enzyme indicators (ALT, AST, GGT)

Within the normal range, liver enzymes such as ALT ≤50 significantly increased the risk of NAFLD (overall population OR = 0.22, males OR = 0.26, females OR = 0.21, all *P* < 0.001). Elevated GGT levels had a similar effect.

##### 3.3.2.5 Combined effect of hyperlipidemia and liver injury

Individuals without dyslipidemia or liver damage had a significantly lower risk of NAFLD (overall population OR = 3.97, males OR = 3.18, females OR = 5.01, all *P* < 0.001). The risk increase was more significant in females.

##### 3.3.2.6 Metabolic indicators

Total Cholesterol (TC), Triglycerides (TG), High-Density Lipoprotein Cholesterol (HDL-C), Low-Density Lipoprotein Cholesterol (LDL-C), Fasting Blood Glucose (FBG), Uric Acid Elevated HDL-C showed the strongest protective effect in females (OR = 5.42, *P* < 0.001), while elevated TG levels were significantly negatively correlated with NAFLD (OR = 0.18–0.26). Hyperuricemia had a particularly significant impact on females (OR = 0.13, *P* < 0.001).

##### 3.3.2.7 Gender differences analysis

In females, the OR values for NAFLD were higher under conditions of dyslipidemia and normal liver function compared to males, suggesting that metabolic disturbances have a more pronounced impact on women. In males, the risk of NAFLD decreased more sharply with age, while the changes in females were relatively gradual, potentially due to endocrine factors.

### 3.4 Multicollinearity assessment (VIF)

The statistical strategy for variable selection in multivariate analysis is primarily focused on controlling multicollinearity. Variables are selected based on correlation coefficients and Variance Inflation Factor (VIF) thresholds. To ensure model stability and precision, variables with high pairwise correlations (e.g., >0.7) and expected VIFs >10, indicating severe multicollinearity, are systematically excluded. The rationale prioritizes comprehensive representative markers, retains continuous variables for greater statistical power, and avoids redundant measures, thereby optimizing the final model for robust interpretation (Table 4).

**Table 4:** Multicollinearity assessment and Variable selection strategy for multivariate analysis.

Variable Category	Selected Variable	Alternative/Variables Considered	Correlation Coefficient	Expected VIF	Selection Rationale
Bilirubin Metabolism	Total Bilirubin	Direct Bilirubin, Indirect Bilirubin	0.920–0.986	50–100+	TBil comprehensively represents bilirubin metabolism; DBil and IBil excluded due to extreme collinearity
Liver Enzymes	ALT	AST, *γ*-GT	0.795–0.460	10–25	ALT retained as primary hepatocellular injury marker; AST excluded due to high correlation
Lipid Profile	Total Cholesterol, HDL-C, TG	LDL-C	0.799 (TC-LDL)	10–20	TC represents global lipid status; HDL-C and TG provide complementary information; LDL-C excluded due to high correlation with TC
Renal Function	Creatinine	BUN	0.408	5–15	Creatinine retained as gold standard; BUN excluded to reduce redundancy
Uric Acid Metabolism	Uric Acid	Hyperuricemia Status	–0.773	8–12	Continuous UA measure preferred over categorical status for greater statistical power
Metabolic Parameters	Fasting Glucose	-	-	2–5	Primary glycemic control marker
Demographic Variables	Age, Gender	-	-	2–6	Fundamental demographic characteristics
Clinical Status	Lipid Status	Dyslipidemia & Liver Injury Status	0.738	8–15	Comprehensive dyslipidemia indicator retained; combined status variable excluded due to overlap

VIF Interpretation: VIF: <5: acceptable multicollinearity; 5–-10: moderate concern; >10: severe multicollinearity requiring variable exclusion. Correlation coefficients represent the strongest pairwise correlation within each variable category.

## 4 Discussion

Fatty liver has become a global health issue, with its increasing prevalence year by year. NAFLD is linked to obesity, metabolic disorders, cardiovascular and cerebrovascular diseases, and a heightened risk of malignant liver tumors, necessitating considerable attention (5, 11). Previous studies indicate that NAFLD prevalence varies by country: 40.2% in South Korea (43), with other rates reported in Japan (44), Indonesia (45), the United States (46), the Netherlands (47), and Italy (48). Japan has the lowest prevalence, while Indonesia has a relatively high prevalence. Fatty liver prevalence is rising in China, especially in southwestern frontier areas with diverse socioeconomic and lifestyle factors. The prevalence of non-alcoholic fatty liver disease (NAFLD) is reported as follows: Chengdu (Southwest) 12.5%, Shanghai (East) 15.0%, Guangdong (South) 17.0%, Central region 24.5% (6), and Chongqing 28.5% (23). The prevalence of NAFLD in China rose from 23.8% (95% CI 16.4%–31.2%) in the early 2000s to 32.9% (95% CI 28.9%–36.8%) by 2018 (20). From a global perspective, it is estimated that China accounts for approximately 49.3% of the worldwide NAFLD cases (49). China is experiencing a fatty liver crisis, with a concerning outlook. Geographically, although fatty liver is highly prevalent across nearly all regions of the country, the prevalence in North China exceeds 50%, while the prevalence in the southern and southwestern regions is approximately 35% (50, 51). However, these data vary due to China's vast territory, age differences, customs, lifestyles, and geographical diversity.

Wenshan, located in southeastern southwestern China bordering Vietnam and Guangxi, provides a valuable setting for studying NAFLD epidemiology across diverse Chinese regions. In this cross-sectional study of 6,419 adults undergoing health checkups in 2022, we found an overall NAFLD prevalence of 33.3% based on ultrasound diagnosis. This rate is higher than those reported in metropolitan areas such as Hong Kong (27%) (52), Chengdu (12.5%) (6), and Shanghai (15.3%) (53), yet lower than the figure from Urumqi in northern China (54.3%) (54). It is comparable to, though slightly higher than, the prevalence in neighboring Chongqing (28.5%) (23). These disparities likely reflect a complex interplay of regional differences in dietary patterns, physical activity, genetic background, and socioeconomic development. Notably, we observed a significant gender disparity, with a much higher prevalence in males (39.6%) than in females (24.1%), a pattern consistent with reports from other regions like Shanghai (55). This underscores that NAFLD is not solely an urban affluent-society issue but is also prevalent in developing border regions, affecting populations with distinct demographic profiles. Our large sample size (*n* = 6,419) provides substantial statistical power for these analyses, and the odds ratios with 95% confidence intervals reported throughout the study robustly quantify the associated risk factors.

The study found that NAFLD prevalence rises with advancing age. Individuals with NAFLD had a significantly higher mean age (47.9 ± 13.3 years) compared to the Non-NAFLD group (43.2 ± 14.2 years) (*P* < 0.001). NAFLD prevalence increased from 14.5% in individuals under 24 to 40.7% in those over 60. In males, the prevalence also showed a steady increase, rising from 17.5% in individuals under 24 years old to 45.60% in the 36–48 age group, and peaking at 48.30% in the 48–60 age group. After 60 years, it declined slightly to 40.20%. For females, prevalence was lowest at 8.10% for those under 24 years, gradually increasing with age to a peak of 41.3% in those over 60 years.

In individuals under 60, NAFLD prevalence was higher in males than females, with a slower increase rate observed in females. Research in South Korea found that NAFLD prevalence was highest among males aged 40–49 and females over 50 (23, 56–58). These findings align with other studies and are consistent with our observations. Studies indicate that NAFLD prevalence rises with age in females, whereas males experience peak prevalence at a younger age. These results align with our findings. The precise causes of this gender disparity warrant deeper discussion. The distinct patterns—higher prevalence in young and middle-aged males and a marked increase in females after 60 years-can be attributed to an interplay of biological and lifestyle factors. This is supported by existing evidence linking the disparity to female physiological traits, such as the potential protective role of estrogen in premenopausal women and decreasing hormone levels (59–62), as well as hypothyroidism (63) and certain lifestyle factors (62, 64).

We found that, aside from total bilirubin (TBil), direct bilirubin (DBil), indirect bilirubin (IBil), alkaline phosphatase (AKP), bile acid (TBA), blood urea nitrogen (BUN), and serum creatinine (CREA), other factor levels were significantly elevated in the NAFLD group compared to the Non-NAFLD group. Factors considered are gender, age, albumin/globulin ratio (A/G), alanine aminotransferase (ALT), aspartate aminotransferase (AST), gamma-glutamyl transferase (GGT), total cholesterol (TC), triglycerides (TG), low-density lipoprotein cholesterol (LDL), fasting blood glucose (FBG), and uric acid (UA). In contrast, high-density lipoprotein cholesterol (HDL) levels were markedly reduced (*P* < 0.001). These findings align with earlier studies (65–68).

Metabolic comorbidities associated with NAFLD encompass obesity, type 2 diabetes, hypertension, metabolic syndrome, and notably, hyperlipidemia, which affects 69.16% (95% CI 49.91%–83.46%) of individuals (5). The NAFLD population showed significantly reduced high-density lipoprotein cholesterol (HDL-C) levels and elevated levels of triglycerides (TG), fasting blood glucose, total cholesterol (TC), low-density lipoprotein cholesterol (LDL-C), and serum uric acid (SUA) (*P* < 0.001) (69). Our study identified a hyperlipidemia prevalence of 63.9% in the NAFLD population, with rates of 69.1% in males and 51.5% in females, consistent with these findings. HDL-C levels were notably elevated in the NAFLD population, with females exhibiting higher levels than males. These findings align with those reported by März W et al. (70) proposed that HDL-C is mainly linked to cardiovascular diseases and advised prioritizing low-density lipoprotein cholesterol (LDL-C) in the context of NAFLD. Our findings appear to support this perspective.

In individuals with NAFLD, total bilirubin (TBIL) levels were notably lower, whereas levels of alanine aminotransferase (ALT), aspartate aminotransferase (AST), gamma-glutamyl transferase (GGT), and serum uric acid (SUA) were significantly elevated (*P* < 0.001) (68, 69). Our study also found consistent results.

In the NAFLD population, variables including Non-NAFLD, Female, Age, TP, GLB, ALB, A/G ratio, ALT, AST, GGT, ALP, TBA, TBil, DBil, IBil, Cr, TC, TG, HDL-C, FBG, and UA exhibited significant associations with liver function status, as indicated by P-values below 0.05, with many being less than 0.001, excluding BUN and LDL-C.

The OR values indicated a positive correlation for Non-NAFLD (2.1), Female (3.85), and HDL-C (2.34). Conversely, laboratory indicators such as ALB, A/G ratio, AST, TBil, DBil, IBil, TC, and TG had OR values <1, indicating a negative correlation. The remaining OR values were close to 1, suggesting weak or negligible correlations (68). Liver injury is primarily characterized by elevated ALT and AST levels, often accompanied by increased GGT (Torres DM) (71, 72). This reinforces the idea that lipid metabolism in non-alcoholic fatty liver disease (NAFLD) indicates an imbalance in liver energy metabolism (Loomba R) (68). Some researchers suggest that abnormal liver function test results may serve as a clue to the presence of NAFLD (Westfall E) (73–75) and should be given attention.

In our multivariate logistic regression analysis, we tested for but did not find significant interaction effects between key variables such as gender and age. Potential multicollinearity among independent variables was assessed and found not to substantially affect the model estimates.

Multivariate logistic regression analysis identified significant associations between lipid status and variables such as Non-NAFLD, Female, Age, TP, GLB, ALB, A/G ratio, ALT, AST, GGT, ALP, TBA, TBil, DBil, IBil, BUN, Cr, TC, TG, HDL-C, LDL-C, FBG, and UA in the NAFLD population, with P-values below 0.05 (many below 0.001). The OR values for Non-NAFLD, Female, A/G Ratio, DBil, and HDL-C were 4.42, 2.72, 1.31, 1.17, and 67.43, respectively, indicating a positive correlation with hyperlipidemia. Conversely, TC, TG, LDL-C, and FBG had OR values <1, indicating a negative correlation. The OR values for the remaining laboratory indicators were close to 1, suggesting minimal or no correlation. Some studies have suggested that dyslipidemia is another metabolic risk factor for NAFLD. Among the NAFLD population, the ratio of triglycerides to high-density lipoprotein cholesterol reached as high as 78%, underscoring the strong link between lipid metabolism abnormalities and NAFLD (73). Research indicates that TG, LDL-C, and HDL-C independently contribute to the risk of NAFLD (23, 76), highlighting their significance.

Excluding Age, GLB, A/G ratio, and BUN, variables such as Non-NAFLD, Female, TP, ALB, ALT, AST, GGT, ALP, TBA, TBil, DBil, IBil, Cr, TC, TG, HDL-C, LDL-C, FBG, and UA demonstrated significant associations with dyslipidemia and liver injury in the NAFLD population, with P-values below 0.05, many under 0.001. The OR values for Non-NAFLD, Female, and HDL-C were 3.97, 5.32, and 22.76, respectively, indicating positive correlations. In contrast, laboratory indicators such as DBil, TC, TG, LDL-C, and FBG had OR values <1, indicating negative correlations. The OR values for other indicators were close to 1, suggesting minimal or no correlation.

Non-NAFLD, Female, and HDL-C exhibited wide confidence intervals across all three conditions, with upper limits significantly greater than 1. There is a strong positive correlation between Non-NAFLD, Female, and HDL-C with hyperlipidemia, liver injury, and the combined condition of hyperlipidemia and non-alcoholic fatty liver disease.

Lipid metabolism is essential in the progression of fatty liver. Dyslipidemia leads to lipid deposition, particularly the accumulation of triglycerides in the liver. This subsequently increases lipid transport, exacerbates hepatic insulin resistance, and ultimately results in the formation of NAFLD (77). Total cholesterol (TC) was not significantly associated with NAFLD in the overall population, contrary to the findings of Xu et al. (78).

In conclusion, the prevalence of NAFLD is steadily increasing due to lifestyle changes. NAFLD is associated with various factors, including hyperlipidemia, liver injury, and the combination of hyperlipidemia and liver injury, all of which represent significant medical challenges to address in the future.

Our study has several limitations that should be considered. First, as a hospital-based cross-sectional study, participation was voluntary, which may introduce selection bias. The cohort likely represents individuals who are more health-conscious or have better healthcare access than the general population, potentially limiting the generalizability of the estimated NAFLD prevalence. Additionally, the cross-sectional design precludes causal inferences between NAFLD and the observed metabolic abnormalities. Second, NAFLD was diagnosed using abdominal ultrasonography. Although this is a practical and widely accepted method in large-scale epidemiology, it is less sensitive than magnetic resonance imaging for detecting mild steatosis and cannot differentiate between simple steatosis and steatohepatitis (NASH). Finally, several potentially important confounders—such as body mass index (BMI), detailed dietary habits, and physical activity levels—were not available in the dataset, limiting our ability to fully adjust for all relevant confounding factors.

Our findings offer concrete insights for shaping NAFLD prevention and management strategies in the Wenshan region. First, given the high prevalence of NAFLD and its strong association with dyslipidemia and liver injury, we recommend integrating routine NAFLD screening using ultrasonography into the management protocols for individuals identified with hyperlipidemia or abnormal liver enzymes in primary care settings. Second, the pronounced gender and age disparities call for targeted public health interventions. Health promotion campaigns focusing on healthy diets and physical activity should be prioritized for middle-aged and older males, who exhibit the highest prevalence, while postmenopausal women should be another key target group for monitoring and education. Finally, these efforts require a multi-sectoral approach, enhancing collaboration between local healthcare providers and public health authorities to raise awareness and allocate resources for the early detection and management of NAFLD.

Our findings offer concrete insights for shaping NAFLD prevention and management strategies in the Wenshan region. First, given the high prevalence of NAFLD and its strong association with dyslipidemia and liver injury, we recommend integrating NAFLD screening (e.g., via ultrasonography) into the clinical evaluation of individuals identified with hyperlipidemia or abnormal liver enzymes in primary care settings. Second, the pronounced gender and age disparities call for targeted public health interventions. Health promotion campaigns focusing on healthy diets and physical activity should be prioritized for middle-aged and older males, who exhibit the highest prevalence, while postmenopausal women should be another key target group for monitoring and education. Finally, these efforts require a multi-sectoral approach, enhancing collaboration between local healthcare providers and public health authorities to raise awareness and allocate resources for the early detection and management of NAFLD.

To address the inherent limitations of this cross-sectional design and to better understand NAFLD progression in this population, several research priorities emerge. As the population continues to age, the burden of metabolic diseases is expected to rise, making it essential to establish prospective longitudinal cohorts in the Wenshan region. Such studies are needed to clarify the temporal and causal relationships between metabolic factors and NAFLD development. Future research should also aim to incorporate a more comprehensive set of variables, including detailed anthropometry (e.g., waist-to-hip ratio), validated dietary and physical activity assessments, smoking history, and socioeconomic indicators. Moreover, integrating non-invasive methods for fibrosis assessment—such as transient elastography (FibroScan) or serum-based fibrosis scores (e.g., FIB-4, NFS)—would greatly enhance the clinical relevance of epidemiological data by enabling risk stratification and early identification of individuals with advanced liver disease. These multi-faceted approaches will be crucial for developing and refining targeted strategies for NAFLD prevention and management in this and similar settings.

## 5 Conclusion

The prevalence of NAFLD is rising globally due to lifestyle changes and metabolic factors, making it an increasing health concern. This research examined the prevalence of NAFLD in healthy adults in Wenshan, southwestern China, and analyzed its association with key risk factors such as hyperlipidemia, liver injury, and their combined impact. The results showed an overall prevalence of 33.30%, with 39.6% in males and 24.1% in females. NAFLD occurred at a younger age in males, while females over 60 years old exhibited a marked increase in prevalence. In individuals under 60, males consistently exhibited a higher prevalence than females.

Multivariate logistic regression analysis identified dyslipidemia and liver injury as significant risk factors for NAFLD. In cases of dyslipidemia, males exhibited a higher risk (male OR > female OR), while under the combined influence of hyperlipidemia and liver injury, females showed a stronger correlation (female OR > male OR). Age, fasting blood glucose, total cholesterol, triglycerides, HDL-C, LDL-C, uric acid, alanine aminotransferase, aspartate aminotransferase, and gamma-glutamyl transferase are closely linked to NAFLD.

The findings highlight the critical role of metabolic disturbances in the development of NAFLD, including hyperlipidemia, liver injury, and their combination. The findings highlight the importance of focused prevention and screening strategies for high-risk groups, especially males, middle-aged individuals, and those with high blood glucose, hyperlipidemia, hyperuricemia, and impaired liver function. Greater attention should also be given to females over 60 years old, given their rising prevalence of NAFLD.

In conclusion, NAFLD is a multifactorial condition requiring further in-depth research to elucidate its pathogenesis and devise effective prevention and management strategies.

## Data Availability

The datasets presented in this study can be found in online repositories. The names of the repository/repositories and accession number(s) can be found in the article/Supplementary Material.
